# Supplementary material for: Outcomes of complex decongestive therapy in managing upper limb lymphedema in female breast cancer patients at a palliative care unit of a tertiary care hospital in Bangladesh
Source: PLoS One. 2025 Jun 20;20(6):e0326040. doi: 10.1371/journal.pone.0326040 (PMC12180636; doi:10.1371/journal.pone.0326040)
Supplement: Attachment — Submitted filename: Response to reviewers.docx [file pone.0326040.s002.docx]

**Outcomes of Complex Decongestive Therapy in Managing Upper Limb Lymphedema in Female Breast Cancer Patients at a Palliative Care Unit of a Tertiary Care Hospital in Bangladesh**

**(Manuscript ID: PONE-D-24-46999)**

**Reviewer#1’s comments to the authors-**

**Comment:**  The aim of this study is to evaluate the effectiveness of CDT for breast cancer patients with upper limb lymphedema and aims to assess the benefits of this treatment despite the challenges and constraints in resource-limited settings. It is well structured in all parts. Only the language must be reviewed.

**Reply:** Thank you. We have revised the language for grammatical errors.

**Reviewer#2 comments to the author-**

**Comment**:  It is not clear in the methodology if the upper limb lymphedema (LE) is unilateral or bilateral. Based on the Results it can be presumed to be bilateral LE.

**Reply:** Thank you. All patients had unilateral lymhedema. The number of affected limbs are mentioned in revised Table 1

**Comment**: How LE was graded as moderate or severe can be mentioned in the Methodology

**Reply 2:** Thank you. We have added the lymphdedema grading in the methods section.

**Changes in the text:**

Line 135-150: Lymphedema grading: The severity of lymphedema in the affected limb was categorized based on circumferential measurements in comparison to the contralateral, unaffected limb [12]-

•	Grade 1 (Mild): Involves distal regions such as the forearm and hand or lower leg and foot. Circumferential difference is less than 4 cm, with no observable tissue changes.

•	Grade 2 (Moderate): Affects the entire limb or the corresponding trunk quadrant, with a circumferential difference of 4–6 cm. Observable tissue alterations such as pitting may occur, and patients may present with erysipelas.

•	Grade 3a (Severe): Lymphedema extends to one limb and its adjacent trunk quadrant, with a circumferential difference exceeding 6 cm. Skin changes including cornification, keratosis, cysts, or fistulae are evident, and recurrent episodes of erysipelas may be reported.

•	Grade 3b (Massive): Characterized by the involvement of two or more extremities, exhibiting the same features as Grade 3a.

•	Grade 4 (Gigantic): Marked by extreme enlargement of the affected limbs, attributed to near-total obstruction of lymphatic flow.

**Comment**: In line 122, it is mentioned that data collection was done between 1 May 2023 and 30 Sep 2023. Does it mean therecruitment was stopped or it implies that the final 6 weeks follow-up post treatment (CDT) was completed for allparticipants on 30 Sep 2023. Rephrase the sentence for better clarity.

**Reply:** Thank you. We revised the line.

**Changes in the text:**

Line 122-124: Participant recruitment commenced on 1 May 2023 and continued through 18 August 2023. The six-week post-treatment follow-up for all enrolled patients was concluded by 30 September 2023.

**Comment**: Lines 162 -165: provide reference

**Reply:** Thank you. The reference has been provided- Ref no 15 and 16

**Comment**: Line 168: What is the interpretation for the color domains mentioned: Normal, red, pink, brown?

**Reply:** Thank you. We have again checked the reference (ref 16) and based on the tool used there we limited the skin integrity domains into two (color change is deleted).

**Comment**: Line 169: Skin thickness can be either normal, thin or thick (stemmer's sign can be mentioned). However, edema can be a separate assessment domain and needn't be mixed with skin thickness.

**Reply:** Thank you. We have corrected the issue. Based on the tool used (ref 16), we added skin edema as separate domain. Skin thickness was actually assed separately, only stemmer’s sign was assed as part of clinical evaluation which was mention in table 5.

**Comment**: Line 177-178: Mention that the CDT was provided for 6 weeks

**Reply:** Thank you. We have corrected the line.

**Changes in the text:**

Line 191-194: All patients underwent intensive CDT phase 1 for 6 weeks during the study period. No alterations to the treatment protocol were made by the investigators. The patients were followed up after 3 weeks during the mid-procedure and after 6 weeks after competition of 6 weeks of CDT.

**Comment**: Lines 197, 200, 202: Ensure either one or two decimal points is provided uniformly in the text as well as in the tables (Table 1 Percentage% check the decimals)

**Reply:** Thank you. We have corrected the lines.

**Comment**: Was there any association between stage of cancer and improvement of LE?

**Reply:** We found no association between cancer stage and improvement of LE

**Comment**: Table 2: Volume of left limb it is mentioned 3505.69+- 3307.87- please recheck value

**Reply:** Thank you. We have corrected the value.

**Comment**: Table 2: the baseline volume for left limb is observed to be greater compared to the right limb. Is it because left limb LE was more common among the participants?

**Reply:** Our participant had mostly left limb LE. The number of affected limb is mentioned in revised Table 1. So the baseline volume for LE was higher in left limb in our study.

**Comment**: Lines 222-223: Can improvement in skin condition be attributed to CDT?

**Reply:** Yes it can be attributed to CDT.

**Changes in the text:**

Line 240-242: However, the improvement in skin condition can be attributed to CDT, as it not only resulted in volume reduction but also included proper skin care

**Comment**: Table 4: Skin thickness and edema can be two separate domains

**Reply:** Skin thickness was not separately measured. So we changed the heading as skin edema in table 4

**Comment**: Lines 262-264: The factors discussed were not assessed in the present study. Its relevance here is not clear

**Reply:** Thank you. As these factors are not assessed in our study, so we deleted the line.

**Comment**: Line 267: Skin thickness and edema to be discussed separately.

**Reply:** Thank you. We made the change accordingly.

**Changes in the text:**

Line 282-289: Regarding skin changes with CDT, we observed a decrease in non-pitting edema over consecutive visits, while pitting edema remained relatively unchanged on the 2nd and 3rd visits. Also regarding skin texture, In terms of skin texture, dryness and shiny skin showed improvement, with nearly half of the patients regaining normal skin by the second and third visits, although these changes were not statistically significant. In contrast, a study in Ireland reported significant improvement in skin texture and decrease in edema [16].

**Comment**: Line 295: Lower limb LE was excluded and not relevant here

**Reply:** Thank you. We deleted the line.

**Comment**: Lines 65 & 301:" early referral..."- the study did not record the delay in accessing treatment (CDT) by patients. Justifythe statement provided here.

**Reply:** Thank you. We made the change accordingly

**Changes in the text:**

Line 65 and 313: Referral of lymphedema patients to specialized clinics and the timely initiation of CDT can significantly reduce ongoing suffering and contribute to overall improvements in quality of life for patients in Bangladesh

**Dear Reviewers, we are grateful for your kind time and substantial review; we believe now the manuscript is more improved, which will satisfy you.**
