# Supplementary material for: Outcomes of complex decongestive therapy in managing upper limb lymphedema in female breast cancer patients at a palliative care unit of a tertiary care hospital in Bangladesh
Source: PLoS One. 2025 Jun 20;20(6):e0326040. doi: 10.1371/journal.pone.0326040 (PMC12180636; doi:10.1371/journal.pone.0326040)
Supplement: Attachment — Submitted filename: Response_to_reviewers_auresp_2.docx [file pone.0326040.s003.docx]

**Outcomes of Complex Decongestive Therapy in Managing Upper Limb Lymphedema in Female Breast Cancer Patients at a Palliative Care Unit of a Tertiary Care Hospital in Bangladesh**

**(Manuscript ID: PONE-D-24-46999.R1)**

**Reviewer#1’s comments to the authors-**

**Comment:**  This study seeks to evaluate the effectiveness of CDT for breast cancer patients with upper limb lymphedema and aims to assess the benefits of this treatment despite the challenges and constraints in resource-limited settings. The paper is well structured but the English must be improved.

**Reply:** Thank you. We have revised the language for grammatical errors.

**Reviewer#2 comments to the author-**

**Comment**:  1. The authors responded that all 42 patients had unilateral (U/L) upper limb lymphedema (LE). Whether U/L LE was one of the inclusion criteria? If so, it may be mentioned in the 'Methods' section in both Abstract and Main text of the manuscript (MS)..

**Reply:** Thank you. We have updated the inclusion criteria.

**Comment**: 2. Line 56: "Significant volume reduction in both right and left upper limbs.." This is quite misleading as all cases had unilateral LE. The sentence shall be reframed for clarity.

**Reply 2:**  Thank you. We have corrected the line.

**Changes in the text:**

Line 56: A significant reduction in the volume of the affected limbs was observed from baseline to the 6th week, as well as from the 3rd week to the 6th week]-

**Comment**: 3. Lines 61-62 and 67, 314: Tightness, heaviness and pain are more subjective responses. It is understood that no

validated scale to assess quality of life (QoL) was used in the study and hence the term QoL may be avoided..

**Reply:**  Thank you. We revised the line.

**Changes in the text:**

Line 65, 324: Timely referral of lymphedema patients to specialized clinics and initiation of CDT can significantly reduce their ongoing suffering in Bangladesh.

**Comment**: 4. Line 125: Correct the spelling of 'massive'

**Reply:** Thank you. The spelling was corrected.

**Comment**: Line 135: Ref 12 has used LE grading by International Society of Lymphology (ISL), the same shall be mentioned in the MS. These grades are found nowhere else other than the Methods. Information on classification of study participants based on these grades needs to be included in the MS. Were these LE grades not considered for the analysis?

**Reply:** Thank you. We updated the reference (ref 12) and added the no of pt according to the grading (table 1). However this grading was only used as inclusion criteria, but not used in analysis.

**Comment**: 6. Line 172: Please correct the tense (Limb volume changes were...).

**Reply:** Thank you. We have corrected the line.

**Comment**: Line 173-174: '...using a measuring tape along with the fixed designated point...' It is not clear. Rephrase the sentence for clarity

**Reply:** Thank you. We have corrected the line.

**Changes in the text:**

Line 172: Limb volume changes were measured by assessing the circumference of the affected limb using a measuring tape at fixed designated points, and the limb volume was then calculated using the truncated cone formula.

**Comment**: Table 1: Write Age (years) with Mean (SD): 50.2+-8.6 and remove years in the rows.)

**Reply:** Thank you. We have made the correction according to your suggestion.

**Comment**: Table 1: Performance status- Correct the decimal points (one decimal) for uniformity. Stage I - III is used which is confusing. Please mention it as the Stage of breast cancer for clarity. Further there is no mention of these stages in the Methods.

**Reply:** We made the correction according to your suggestion (table 1).

**Comment**: Table 2: Correct decimal points for the volume mentioned (2492.4981, 2691.70 etc...)

**Reply:** Thank you. We have corrected the values.

**Comment**: Line 230-231: Table 3 information cited doesn't match with the sentence provided.

**Reply:** correction was made accordingly.

**Comment**: Table 3, 4: Title and information provided in tables do not match

**Reply:** Table titles are corrected (table 3, 4)

**Comment**: Table 3: Ensure uniform font size

**Reply:** Text size revised.

**Comment**: Lines 236-243: Table 4 is cited wrongly in the text

**Reply:** Thank you. We made the suggested corrections.

**Comment**: Tightness, heaviness and pain...these are more subjective symptoms and supposedly assessed orally(using questionnaire). It may be clarified that these symptoms were self-reported perceptions by the patients.

**Reply:** Thank you. We made added ‘self-reported symptoms and observed signs’ in the title of table 5.

**Comment**: Line 258: Check for grammatical error

**Reply:** Thank you. The line has been checked and corrected.

**Comment**: Correct the decimal points.

**Reply:** Thank you. Suggested corrections are made.

**Comment**: Previous comment: The authors have replied that no association was found between stage of cancer and improvement of LE. The same shall be mentioned in the Results. Also was there any association found between the LE grades and improvement in LE? If so, please mention in the MS..

**Reply:** Thank you. Suggested lines are mentioned in the result. As we used lymphedema grading only as inclusion criteria, but didn’t included in the analysis, so we didn’t actually assessed the change in lymphedema grade with volume change

**Changes in the text:**

Line 172: However, we did not find any association between cancer stage and the volume reduction of the affected limb

**Dear Reviewers, we are grateful for your kind time and substantial review; we believe now the manuscript is more improved, which will satisfy you.**
